# Health trajectories before initiation of non-invasive ventilation for chronic obstructive pulmonary disease: a French nationwide database analysis

**DOI:** 10.1016/j.lanepe.2023.100717

**Published:** 2023-08-29

**Authors:** Jean-Louis Pepin, Pauline Lemeille, Hélène Denis, Anne Josseran, Florent Lavergne, Arnaud Panes, Sébastien Bailly, Alain Palot, Arnaud Prigent, the medXcloud group

**Affiliations:** a University Grenoble Alpes, Inserm U1300, CHU Grenoble Alpes, HP2, Grenoble, France; b HEVA, Lyon, France; c ResMed Science Center, Saint-Priest, France; d Hôpital Saint-Joseph, Marseille, France; e Polyclinique Saint-Laurent, Rennes, France

**Keywords:** Chronic obstructive pulmonary disease, Non-invasive ventilation, Health trajectories, Comorbidities, Health database

## Abstract

**Background:**

Chronic obstructive pulmonary disease (COPD) is the most common indication for long-term domiciliary non-invasive ventilation (NIV) but there is uncertainty in data supporting current guidelines. This study described health trajectories before initiation of at-home NIV in people with COPD, and compared mortality outcomes between groups with different pre-NIV health trajectories.

**Methods:**

Data were from the French national health insurance reimbursement system database for individuals with COPD aged ≥40 years and ≥1 reimbursement for NIV between 1 January 2015 and 31 December 2019. Common health trajectories were determined using time sequence analysis through K-clustering (TAK analysis).

**Findings:**

Data from 54,545 individuals were analysed; the population was elderly (median age 70 years) with multiple comorbidities. Four clusters were generated. Cluster 1 (n = 35,975/54,545; 66%) had NIV initiated in ambulatory settings or after the first acute event/exacerbation. Cluster 2 (6653/54,545; 12%) started NIV after ≥2 severe exacerbations in the previous 6 months. Cluster 3 (11,375/54,545; 21%) started NIV after frequent severe COPD-related exacerbations in the previous year. Cluster 4 (652/54,545; 1%) started NIV after many long-lasting severe exacerbations. The four clusters differed in age, sex, comorbidities, pre-NIV investigations, and prescriber/location of NIV initiation. Mortality differed significantly between clusters: highest in Cluster 4 and lowest in Cluster 1.

**Interpretation:**

The significant heterogeneity in clinical initiation of NIV probably reflects the current lack of strong evidence and guideline recommendations. Knowledge about the characteristics and outcomes in different clusters should be used to address inequities and facilitate more consistent and personalised use domiciliary NIV in COPD.

**Funding:**

JLP and SB are supported by the 10.13039/501100001665French National Research Agency in the framework of the “Investissements d’avenir” program (ANR-15-IDEX-02) and the “e-health and integrated care and trajectories medicine and MIAI artificial intelligence (ANR-19-P3IA-0003)” Chairs of excellence from the 10.13039/100019979Grenoble Alpes University Foundation. This work was supported by 10.13039/100017647ResMed.

**Research in context**

**Evidence before this study**

According to the latest Global Burden of Disease data, chronic obstructive pulmonary disease (COPD) is a highly prevalent chronic medical condition. In addition, COPD is the third leading cause of death worldwide. Many individuals with severe COPD and hypercapnic respiratory failure require long-term at-home non-invasive ventilation (NIV), and COPD is the most common indication for this therapy. Although both the European Respiratory Society (Ergan et al. Eur Respir J 2019; 54: 1901003) and American Thoracic Society (Macrea et al. AJRCCM 2020; 202:e74-87) have guidelines on the use of NIV in COPD, there is a high degree of uncertainty in the data supporting current recommendations and there is still a debate around whether and for which COPD subtypes NIV should be continued over the long-term at home after its usage in the post-acute setting. In addition, the optimal timing for initiation of NIV therapy after recovery from an acute exacerbation of COPD is not yet known.

**Added value of this study**

The French national health insurance reimbursement system database used in this study is a large unbiased, well anonymised claims database that covers >99% of the French population. This means that study data are robust and reflect population-wide practices. These data address a current gap in the literature because there is also a lack of real-world evidence regarding health trajectories prior to the initiation of at-home NIV in individuals with COPD. The identification of four distinct groups showing quite different pre-NIV health trajectories, and the finding of different mortality rates in these groups, could be used to reduce health inequities and facilitate a more personalised and evidence-based approach to the use of NIV in stable COPD.

**Implications of all the available evidence**

Prescription of at-home NIV in individuals with COPD occurs at a variety of different stages in the disease trajectory. The different approaches used in the four clusters identified in this analysis likely reflect the current lack of consistent evidence and consensus with respect to guideline recommendations for NIV set-up and initiation.

## Introduction

Chronic obstructive pulmonary disease (COPD) is a common respiratory condition that is characterised by gradual worsening of symptoms and lung function. Worldwide in 2019 there were 202.3 million prevalent cases of COPD, 3.3 million COPD-related deaths, and COPD was responsible for the loss of 74.4 million disability-adjusted life-years.^1^ The global age-standardised prevalence of COPD is 2638 per 100,000 population.^1^ Although these statistics alone are sobering, available data suggest that underdiagnosis is also a significant issue, meaning that a large number of individuals who have undiagnosed COPD may not be appropriately treated.^2^ Overall, COPD is now a global health crisis.^3^

Acute exacerbations are a key feature of COPD, and these become increasingly common as the disease progresses. In addition, the occurrence of acute exacerbations of COPD (AECOPD) is an important driver of the increased economic burden associated with this disease.^4^^,^^5^ COPD also negatively impacts on physical, mental and social status.^6^ In addition, it is widely accepted that COPD symptoms have a substantial detrimental impact on health status and quality of life.^7^

Many individuals with severe COPD and hypercapnic respiratory failure require long-term at-home non-invasive ventilation (NIV). In fact, COPD is the most common indication for long-term domiciliary NIV, followed by obesity hypoventilation syndrome.^8^^,^^9^ However, there remain a number of unanswered questions regarding long-term at-home NIV for the management of COPD.^10^, ^11^, ^12^ Guidelines from the European Respiratory Society (ERS) and the American Thoracic Society (ATS) describe the indications for long-term NIV in individuals with COPD, both for long-term therapy and for shorter-term support after an acute exacerbation (post-acute).^10^^,^^11^ However, studies supporting current recommendations have included highly selected groups of individuals with COPD that are not representative of the clinical heterogeneity of COPD. This means that current recommendations do not take into account the multiplicity of COPD manifestations. Therefore, there is still a debate around whether, and for which COPD subtypes, NIV should be continued over the long-term at home after its usage in the post-acute setting.^13^ Furthermore, although chronic NIV appears to be beneficial for individuals with persistent hypercapnia after recovery from a COPD exacerbation, the optimal timing of therapy is not yet known.^12^ There is also a lack of real-world evidence regarding health trajectories prior to the initiation of at-home NIV in individuals with COPD, including the impact of comorbidities, rate of previous AECOPD, and prescribers. This highlights the need to identify different health trajectories before the initiation of at-home NIV.

This analysis used data from the French national health insurance reimbursement system database (“Système national des données de santé”; SNDS) – a world-class database that covers 99% of the French health system—to describe health trajectories before the initiation of at-home NIV in people with COPD, and determine whether different health trajectories are associated with different mortality outcomes. The hypothesis was that specific health trajectories before NIV initiation would be representative of different COPD phenotypes/clinical subtypes, prescriber behaviours, and health care system organisation.

## Methods

### Study design and data source

Data for this observational study were extracted from the SNDS.^14^ In brief, the SNDS covers 98.8% of the population living in France and collects individual pseudonymised data from beneficiaries of almost all health insurance schemes. Data availability varies by year and scheme. Therefore, for consistency throughout the study period, we restricted data extraction to the general scheme (for private sector employees), covering approximately 76% of the French population (around 40 million adults). Retirees, unemployed people, and welfare beneficiaries are covered by the scheme corresponding to their former occupation.

The SNDS contains socio-demographic data (age, sex, and area), and information on all health care expenses, including outpatient visits, reimbursed medication, medical procedures, hospital admission diagnoses and procedures, and date of death. Although diagnoses and purpose of outpatient visits, and the results of laboratory tests or other examinations, are not available, the SNDS contains a list of 30 long-term chronic diseases coded according to the International Classification of Diseases 10th revision (ICD-10), for which patients are granted full reimbursement for disease-related healthcare expenses.

Specific approvals for this study were obtained from the Comité éthique et scientifique pour les recherches, les études et les évaluations dans le domaine de la santé (CESREES; ref: 3904033) and from the “Commission Nationale Informatique et Liberté” (CNIL), the French information technology and personal data protection authority (DR 2021 162 and n° 921,198).

### Study population

The study population comprised individuals aged ≥40 years who had at least one reimbursement for NIV between 1 January 2015 and 31 December 2019. Only incident cases were included (i.e. those with no NIV treatment in the previous 5 years); the index date was the date of first delivery of NIV during the study inclusion period, and the 5-year period was chosen to provide a sufficiently wide time window to identify and exclude individuals previously treated with NIV. The presence of COPD was determined based on ICD-10 codes and treatment with long-acting β-agonist/long-acting muscarinic antagonist combinations and inhaled corticosteroids before or during treatment with NIV (see Supplementary Methods for full details). Individuals with a diagnosis of cystic fibrosis or any neuromuscular disease were excluded, but those with asthma and/or other respiratory diseases other than COPD remained eligible. Data collection for included individuals continued until December 2020.

### Outcomes definition

Only severe exacerbations of COPD that required hospitalisation were recorded. These were identified using the published algorithm validated by the French Institute for Public Health Surveillance (InVS). InVS serves as reference governance body for methodology of studies based on the French health insurance database in France. The algorithm included the different ICD-10 diagnoses and their prioritised score as principal diagnosis, related diagnosis and/or associated diagnosis to have an accurate characterization of severe COPD exacerbations (see Supplementary Methods in the online supplement for full details). This algorithm has been used in previous studies that utilised SNDS data.^15^^,^^16^ Hospitalisations were split into two groups: hospitalisations in respiratory ward, cardiology or intensive care units, and other types of hospitalisations. Only hospitalisations for cardiology or respiratory diagnoses were recorded. Claims in the five years preceding NIV initiation were used to identify comorbidities (based on ICD-10 codes and medication use) and relevant medical history, and to confirm incident status.

### Statistical analysis

**Fig. 1:**
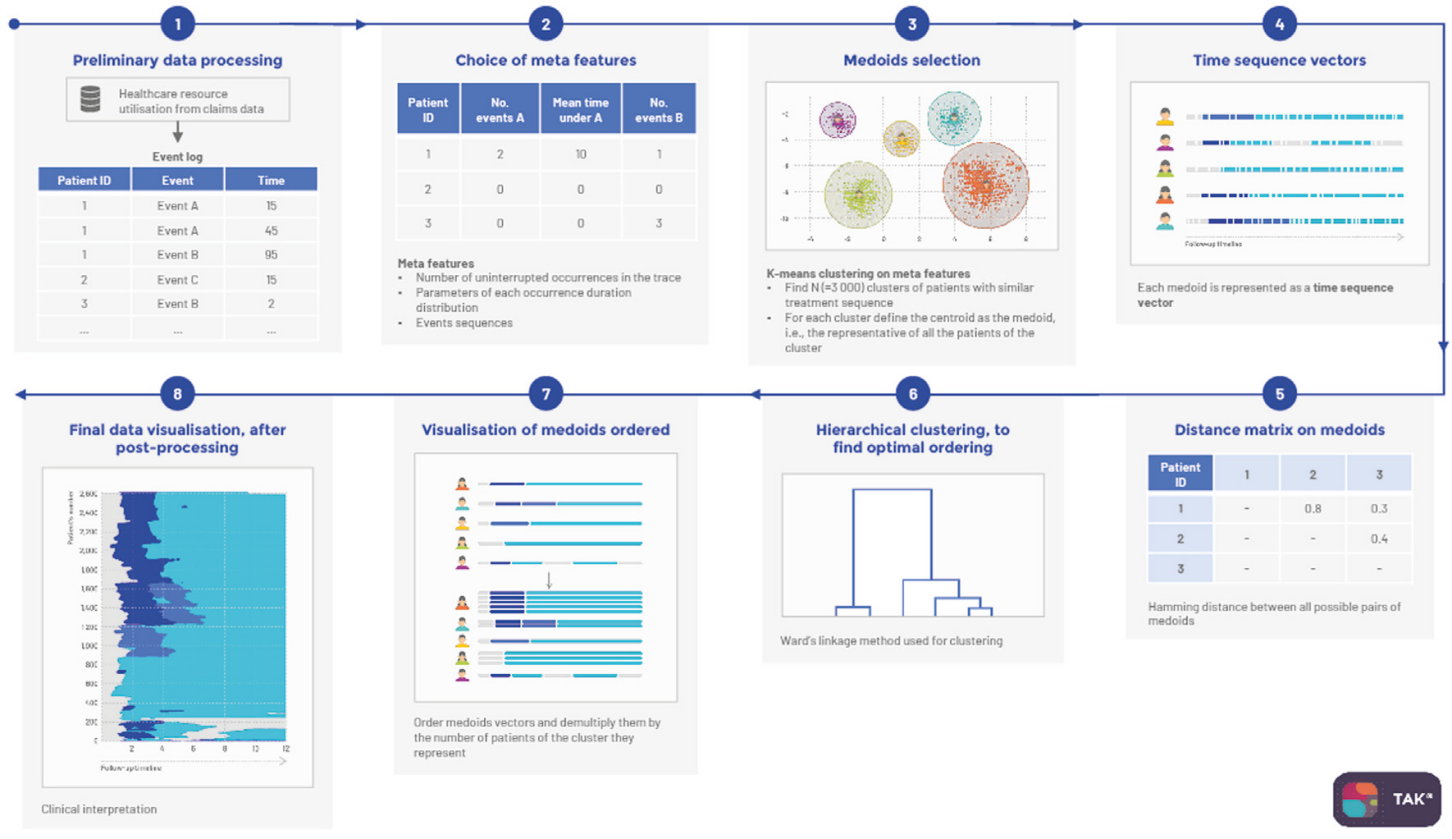
**TAK-medoid methodology**.

Data on the study population are presented using descriptive statistics (median and interquartile range for quantitative variables, and frequency and percentage for qualitative variables). Machine learning, time sequence analysis through K clustering (TAK analysis) (Fig. 1)^17^^,^^18^ was used to identify clusters of participants who had similar COPD-related health trajectories in the year prior to initiation of NIV.

For computational and visualisation purposes, the TAK-medoids algorithm applied hierarchical agglomerative clustering on “patient-medoids”. Each patient-medoid represents one group of individuals who had similar health trajectories. These patient-medoids are obtained after the application of a K-means algorithm^19^ based on specific meta-features (i.e. number of days description) related to each of the health events of interest (hospitalisation in the intensive care unit, hospitalisation for cardiology or respiratory diagnosis, and hospitalisation for COPD exacerbations). The meta-features definition and the K number of patient-medoids are chosen so that the Ward’s distance between a patient-medoid and all the patients it represented was small (i.e. high homogeneity score between patients) (steps 1–3). The TAK algorithm was applied on patient-medoids, which ordered the most similar sequences of health trajectories using an unsupervised hierarchical agglomerative clustering method^20^ (steps 4–6). First, a matrix of distances between all possible pairs of patient-medoids was computed; the distance used was the Hamming distance.^21^ Next, Ward’s linkage method^20^ was used to combine the closest pairs of patients into groups (clusters). Finally, to achieve better visual outputs, every patient-medoid (represented as a time vector) was multiplied by the number of patients it represented (the assumption being that they were similar enough to all be represented by the same patient-medoid on the final image) (steps 7–8). The final number of clusters was determined based on clinical relevance (using input from medical experts) and internal validation was assessed using the Silhouette index.^22^ Additional details relating to the appropriateness of TAK analysis in this setting are provided in the Supplementary Methods. The presentation of clusters was ordered from least severe (no/few exacerbations; Cluster 1) to most severe (many exacerbations; Cluster 4). Differences between the final clusters were determined using a Chi-square test for categorical variables and one-way analysis of variance for continuous variables.

Survival curves for the four different clusters were visualised using the Kaplan Meier method. Differences between the survival curves for different clusters were determined using log-rank tests. In addition, multivariable Cox regression was computed to evaluate mortality risk in different patient subgroups (by age, sex, Charlson comorbidity score and patient cluster).

### Role of the funding source

Representatives of the study sponsor were involved in the design of the study. The first draft of the manuscript was prepared by JLP with the assistance of an independent medical writer funded by ResMed. The manuscript was reviewed and edited by all the authors. All authors made the decision to submit the manuscript for publication and assume responsibility for the accuracy and completeness of the analyses and for the fidelity of this report to the trial protocol.

## Results

### Study population

A total of 54,545 incident patients initiated on long-term NIV were included in the analysis; a similar number of individuals was included during each year of the study (Supplementary Fig. S1). Overall, the study population was elderly with multiple comorbidities (most commonly hypertension/cardiovascular disease) (Table 1). The first prescriber of NIV was a hospital practitioner in the majority of participants (n = 35,387; 65%), followed by respiratory physicians in private practice (n = 14,069; 26%).

**Table 1:** Baseline demographics and characteristics of the study population.

	Participants (n = 54,545)
Age, years	70 (62, 79)
Male sex, n (%)	27,920 (51)
Number of exacerbations requiring hospitalisation in the year before NIV initiation	1 (0, 2)
Duration of hospitalisation during AECOPD, days	11 (6, 20)
Medical history within the previous 5 years, n (%)	
Undernutrition	15,140 (28)
Morbid obesity	25,158 (46)
Sleep apnoea	18,309 (34)
Hypertension or cardiovascular disease	41,262 (76)
Diabetes	16,137 (30)
Psychiatric diseases, anxiety or depression	20,338 (37)
Charlson comorbidity index n (%)	
0	3889 (7)
1–2	42,572 (78)
3–4	7591 (14)
≥5	493 (1)

Values are median (interquartile range) or number of patients (%).

AECOPD, acute exacerbation of chronic obstructive pulmonary disease; NIV, non-invasive ventilation.

### Health system interactions in the year before NIV

Study participants had a high number of general practitioner (GP) consultations in the year before NIV (median 9, interquartile range 5–14), and 70–75% of the population was referred to a hospital specialist and/or private practice respiratory physician (Table 2). Pulmonary function tests and sleep studies were performed by 63% and 25% of participants, respectively (Table 2).

**Table 2:** Investigations and consultations in the year before the initiation of non-invasive ventilation, for the total cohort and by cluster.

	Total cohort (n = 54,545)	Cluster				
		1 (n = 35,975)	2 (n = 6543)	3 (n = 11,375)	4 (n = 652)	p-value
Sleep studies (PG or PSG)						
0	40,765 (75)	26,804 (75)	4883 (75)	8519 (75)	559 (86)	<0.001
1–2	12,516 (23)	8403 (23)	1494 (23)	2538 (22)	81 (12)	<0.001
≥3	1264 (2)	768 (2)	166 (3)	318 (3)	12 (2)	<0.001
Pulmonary function tests						
0	20,335 (37)	14,463 (40)	2144 (33)	3515 (31)	213 (33)	<0.001
1–2	25,624 (47)	17,277 (48)	3036 (46)	5066 (45)	245 (38)	<0.001
3–5	7313 (13)	3804 (11)	1130 (17)	2254 (20)	125 (19)	<0.001
≥6	1273 (2)	431 (1)	233 (4)	540 (5)	69 (11)	<0.001
Hospital physician consultations						
0	24,442 (45)	18,487 (51)	2297 (35)	3461 (30)	197 (30)	<0.001
1–2	14,943 (27)	9325 (26)	2011 (31)	3399 (30)	208 (32)	<0.001
3–5	8836 (16)	4997 (14)	1257 (19)	2447 (22)	135 (21)	<0.001
≥6	6324 (12)	3166 (9)	978 (15)	2068 (18)	112 (7)	<0.001
Pulmonologist consultations						
0	47,572 (87)	31,647 (88)	5699 (87)	9659 (85)	567 (87)	<0.001
1–2	5930 (11)	3775 (10)	685 (10)	1400 (12)	70 (11)	<0.001
≥3	1043 (2)	553 (2)	159 (2)	316 (2)	15 (2)	<0.001
GP consultations						
0	2491 (5)	1685 (5)	279 (4)	474 (4)	53 (8)	<0.001
1–2	3106 (6)	2219 (6)	305 (5)	484 (4)	98 (15)	<0.001
3–5	9960 (18)	7420 (21)	998 (15)	1394 (12)	148 (23)	<0.001
6–10	17,602 (32)	11,973 (33)	2083 (32)	3370 (30)	176 (27)	<0.001
11-20	16,816 (31)	10,362 (29)	2225 (34)	4104 (36)	125 (19)	<0.001
>20	4570 (8)	2316 (6)	653 (10)	1549 (14)	52 (8)	<0.001

Values are number of patients (%).

GP, general practitioner; PG, polygraphy; PSG, polysomnography.

### Health trajectory cluster groups

A total of 3000 patient-medoids were identified using the K-means clustering algorithm. The mean homogeneity score for the distance between patient-medoids and patients they represent was 95.3% (standard deviation 8.0%). The total cohort was divided into four clusters based on visualisation of observed trajectories in the year prior to initiation of NIV.

#### Cluster 1

**Fig. 2:**
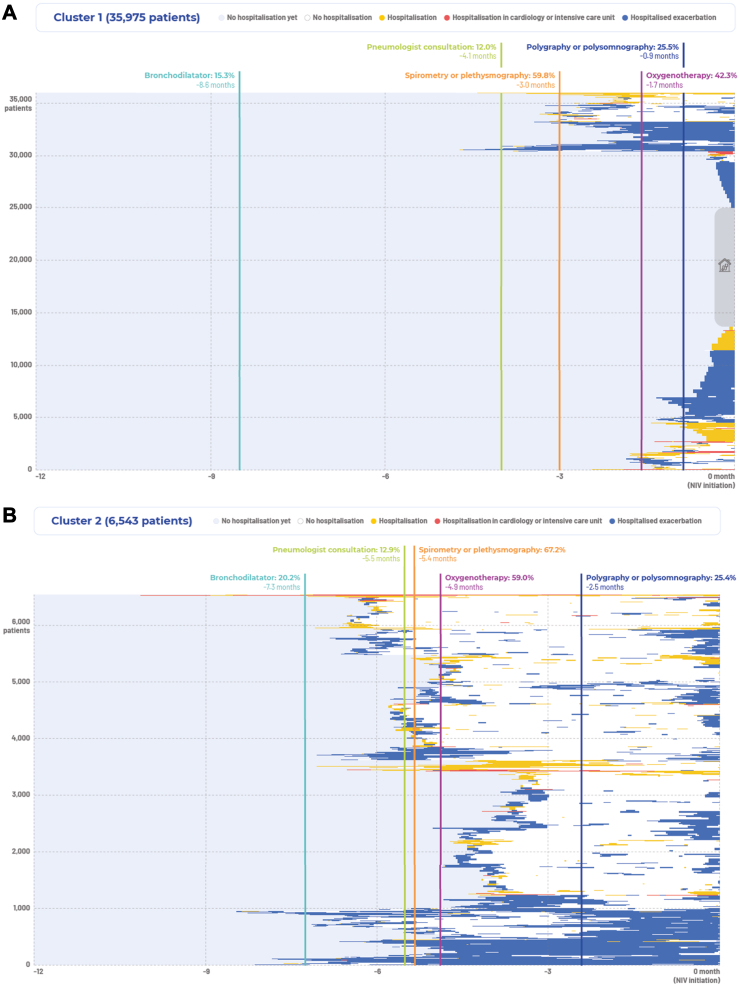

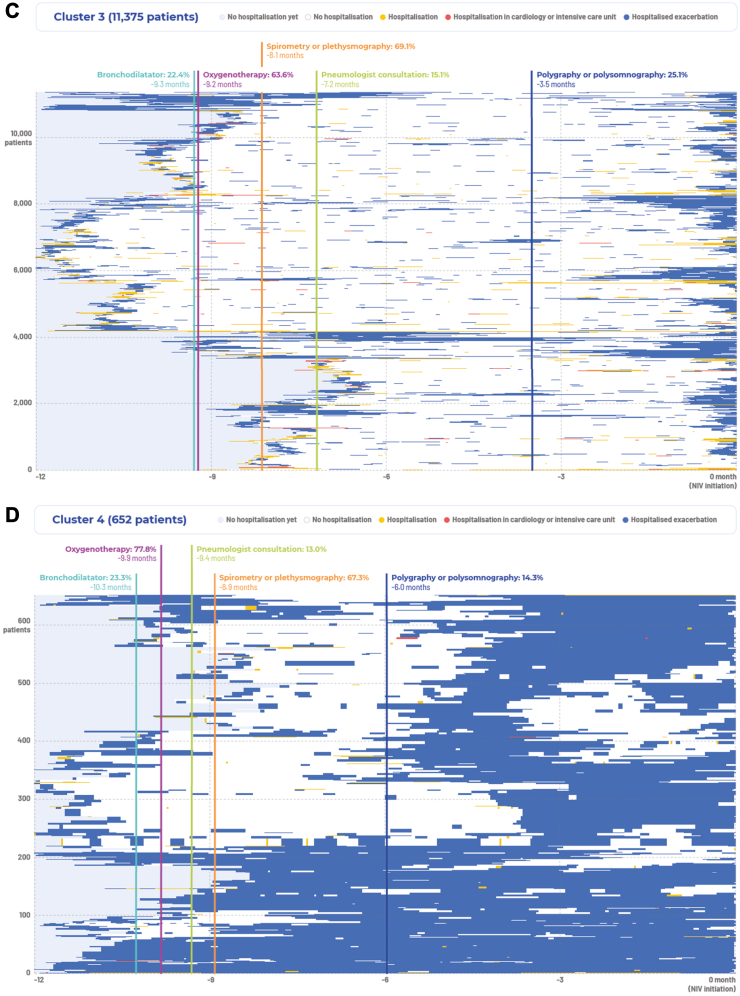
**Trajectories in the year before initiation of at-home non-invasive ventilation by patient cluster.** The end of the trajectory is the initiation of non-invasive ventilation (NIV); the grey rectangle with a house symbol in Cluster 1 indicates individuals who had initiation of NIV at home or during private practice consultation (ambulatory settings) without any prior hospitalisation or exacerbation (n = 11,405; 21%); vertical lines and associated text indicate proportion of participants and median timing of treatments and procedures prior to NIV initiation. (**A**) Trajectories in the year before initiation of at-home non-invasive ventilation in cluster 1. (**B**) Trajectories in the year before initiation of at-home non-invasive ventilation in cluster 2. (**C**) Trajectories in the year before initiation of at-home non-invasive ventilation in cluster 3. (**D**) Trajectories in the year before initiation of at-home non-invasive ventilation in cluster 4.

NIV was started in ambulatory settings or after no more than one hospitalisation for a severe acute event/exacerbation (Fig. 2A). This was the largest cluster (n = 35,975; 66%). These individuals were not clearly identified as having COPD before experiencing an exacerbation or hospitalisation in the months preceding NIV initiation. A small proportion (15%) were prescribed bronchodilators, but pulmonary function tests (in 59.8%) and sleep studies (in 25.5%) tended to take place closer to the time of NIV initiation (median 3 months prior to the start of NIV). Oxygen therapy preceded NIV in 42% of cases (median 1.7 months before NIV).

There were 11,405 individuals (21% of the total cohort) who had NIV initiated at home or during private practice consultations without any hospitalisation or exacerbation in the year before NIV was started. These patients were younger, had fewer comorbidities, and were more likely to have sleep apnoea compared with the overall study cohort (Supplementary Table S1). In addition, this subgroup had NIV prescribed by respiratory physicians in private practice more often, were more likely to be investigated for sleep-disordered breathing, and were less likely to receive oxygen therapy before NIV (Supplementary Table S2).

#### Cluster 2

The initiation of NIV took place after at least two hospitalisations for severe exacerbations in the previous 6 months (n = 6543; 12%) (Fig. 2B). These individuals had an initial diagnosis of COPD or confirmation of a diagnosis during a first health event, had undergone frequent pulmonary function tests and sleep studies (in 67.2% and 25.4% at a median of 5.4 and 2.5 months, respectively, prior to NIV initiation), and were sometimes prescribed a bronchodilator (20.2% of individuals) and oxygen (59.0%) therapy (at median 7.3 and 4.9 months before NIV, respectively). Initiation of NIV generally came after a second health event or a long-lasting exacerbation (the latter was defined as an exacerbation lasting more than one week^23^).

#### Cluster 3

NIV was started after frequent hospitalisations for severe COPD exacerbations in the previous year (n = 11,375; 21%), usually after more than 2 or 3 separate health events (Fig. 2C). Lung function testing (median 8.1 months before NIV) and oxygen therapy (median 9.2 months before NIV) were used in approximately two-thirds of individuals in this cluster.

#### Cluster 4

In this small subgroup of individuals (n = 652; 1%), NIV was initiated after many long-lasting hospitalisations (Fig. 2D). Specialised care and follow-up appeared to be underutilised, with only 13.0% having a pulmonologist consultation, although most (77.8%) were on oxygen therapy and had undergone pulmonary function testing (67.3%).

### Differences between clusters

There were significant differences between clusters across most variables evaluated in this analysis. Regarding investigations and consultations in the year before NIV, sleep studies were least common and pulmonary function tests most common in Cluster 4, hospital physician consultations occurred more often in Cluster 4, and individuals in Cluster 1 had the highest number of GP consultations (Table 2). The proportion of males, number of hospitalisations for exacerbations in the year before NIV, and number of comorbidities all increased from Cluster 1 to Cluster 4 (Table 3). The majority of comorbidities were most common in Cluster 4 and least common in Cluster 1, except for sleep apnoea and obesity where the reverse was the case (Table 3). There was no significant difference between clusters regarding the prescriber of first NIV therapy. However, NIV initiation was more likely to occur in hospital for individuals in Cluster 4 compared with the overall cohort (74% vs. 65%) and less likely to be undertaken by a pneumonologist in private practice (19% vs. 26%).

**Table 3:** Baseline demographics and characteristics of the study population, by cluster.

	Cluster				
	1 (n = 35,975)	2 (n = 6543)	3 (n = 11,375)	4 (n = 652)	p-value
Age, years	69 (62, 79)	72 (64, 81)	71 (63, 80)	71 (63, 80)	<0.001
Male sex, n (%)	17,989 (50)	3440 (53)	6127 (54)	364 (56)	<0.001
Exacerbations requiring hospitalisation in the year before NIV initiation					
Total number	0 (0, 1)	2 (0, 2)	2 (1, 3)	3 (2, 4)	<0.001
0, n (%)	3214 (9)	323 (5)	340 (3)	12 (2)	<0.001
1–2, n (%)	28,904 (80)	4907 (75)	8299 (73)	462 (71)	<0.001
3–4, n (%)	3727 (10)	1207 (18)	2507 (22)	150 (23)	<0.001
≥5, n (%)	130 (<1)	106 (2)	229 (2)	28 (4)	<0.001
Duration of hospitalisation during AECOPD, days	12 (7, 21)	12 (6, 22)	10 (6, 17)	22 (8, 73)	<0.001
Medical history within the previous 5 years, n (%)					
Undernutrition	7840 (22)	2527 (39)	4341 (38)	432 (66)	<0.001
Morbid obesity	16,604 (46)	3000 (46)	5304 (47)	250 (38)	<0.001
Sleep apnoea	11,827 (33)	2252 (34)	4048 (36)	182 (28)	<0.001
Hypertension or cardiovascular disease	25,776 (72)	5450 (83)	9494 (83)	542 (83)	<0.001
Diabetes	10,514 (29)	1918 (29)	4341 (38)	432 (66)	<0.001
Psychiatric diseases, anxiety or depression	11,923 (33)	2820 (43)	5192 (46)	403 (62)	<0.001
Charlson comorbidity index n (%)					
0	3214 (9)	323 (5)	340 (3)	12 (2)	<0.001
1–2	28,904 (80)	4907 (75)	8299 (73)	462 (71)	<0.001
3–4	3727 (10)	1207 (18)	2507 (22)	150 (23)	<0.001
≥5	130 (<1)	106 (2)	229 (2)	28 (4)	<0.001

Values are median (interquartile range) or number of patients (%).

AECOPD, acute exacerbation of chronic obstructive pulmonary disease; NIV, non-invasive ventilation.

### Mortality by patient cluster

**Fig. 3:**
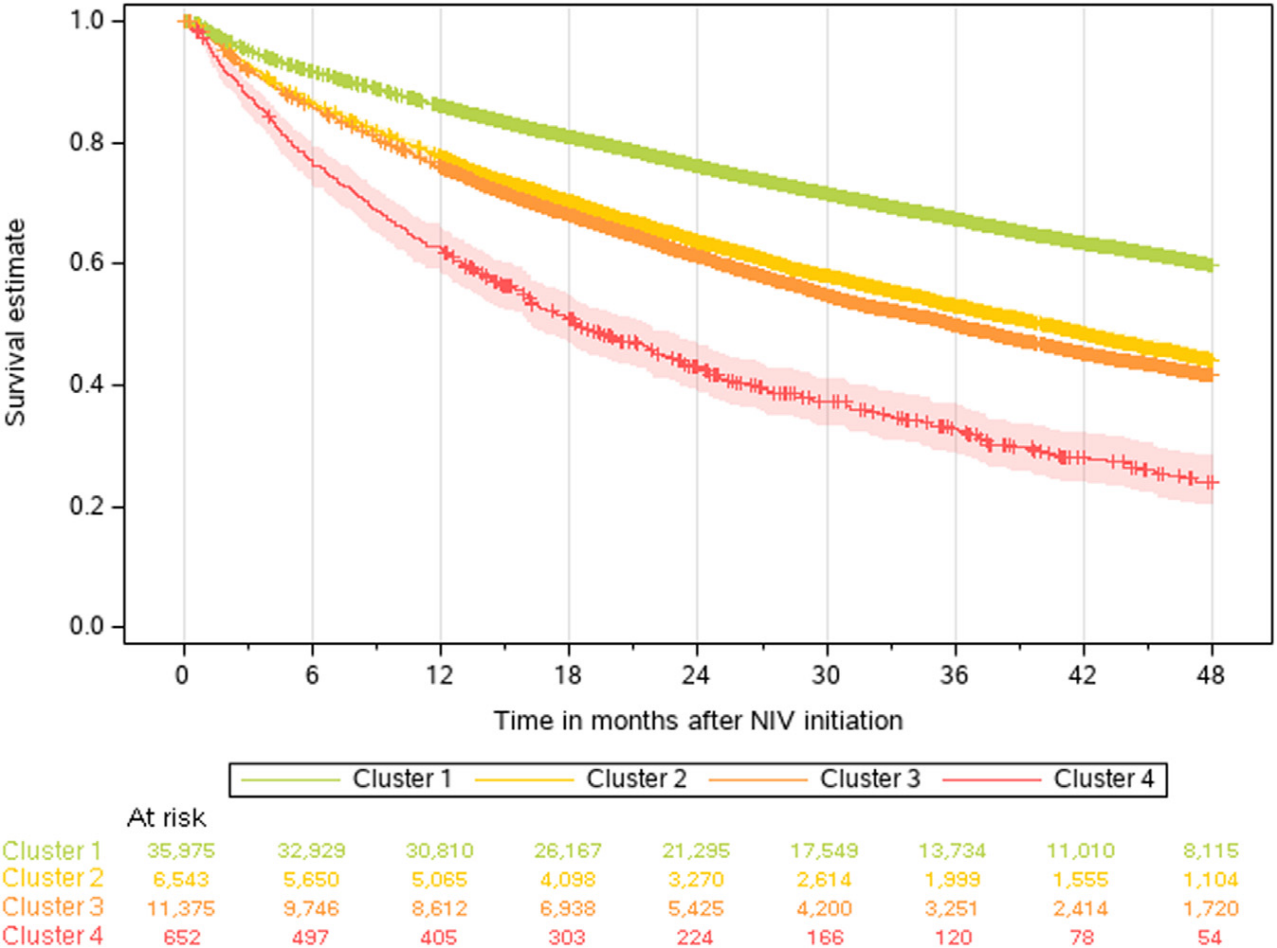
**Kaplan Meier survival curves by patient cluster.** NIV, non-invasive ventilation.

Kaplan–Meier curves showed significant differences in mortality between patient clusters (Fig. 3). Mortality was highest in Cluster 4, lowest in Cluster 1, and intermediate in Clusters 2 and 3. Pairwise comparisons between the different clusters showed significantly greater mortality risk in Clusters 2, 3 and 4 compared with Cluster 1, in Clusters 3 and 4 versus Cluster 2, and in Cluster 4 versus Cluster 3 (p ≤ 0.0001 for all comparisons) (Supplementary Table S3). Mortality risk in patient subgroups (multivariable Cox regression analysis) is shown in Supplementary Table S4.

## Discussion

These data address a current lack of evidence relating to health trajectories prior to the start of home NIV using a unique dataset that included all patients started on long-term NIV in France between 2015 and 2019. There were four distinct health trajectories before the initiation of NIV. Factors that differed between the four health trajectory clusters included patient factors such as age, sex and comorbidities, healthcare factors such as investigations prior to NIV, and the prescriber and location of NIV initiation. The lack of both consensus and strong evidence to support current recommendations for NIV set-up and initiation^10^, ^11^, ^12^ and the multiple different COPD phenotypes could explain the variety of different health trajectories seen before the start of NIV therapy in this group of French individuals with COPD. Another key finding of the study was the significantly different mortality rates between clusters.

The largest group of individuals in this study were grouped into Cluster 1 (two-thirds of the total sample), and were started on NIV in ambulatory settings or after the first hospitalisation for a severe acute event/exacerbation. These individuals were younger, had fewer comorbidities, were less likely to be undernourished, and were more likely to have undergone evaluations to detect sleep-disordered breathing. It could be hypothesised that the latter indicates an endotype with obesity (or sarcopenia obesity). The large number of patients in Cluster 1 who were started on NIV within a few weeks after first visiting a respiratory physician provides an indication of the high rates of underdiagnosis of COPD, whereby a significant proportion of individuals living with COPD have not been identified as having the disease.^2^ Furthermore, the low rates of bronchodilator use in this cluster likely reflect the fact that a high proportion were diagnosed with COPD just before initiation of NIV and would have had the addition of these medications at a later point in their care pathway.

A notable feature of Cluster 3 was that use of supplemental oxygen therapy was common. This was not due to older age because age did not differ from that in other clusters, and palliative care was excluded. Therefore, we hypothesise that this reflects the characteristic of this cluster as having been started on NIV after several AECOPD, increasing the likelihood that oxygen therapy was prescribed before consideration of NIV.

Cluster 4 represents the sickest population, who had delayed access to care and were probably less good at utilising healthcare systems. Our dataset did not include information on ethnicity or poverty, but we can speculate that geographic and socioeconomic differences were present. Even in Europe, COPD is a disease of poverty. Differences in coverage between insurers disproportionally impact non-White and low socioeconomic status individuals with COPD. This needs to be formally recognised by inter-professional, multidisciplinary committees to advocate for policies that reduce inequities and align with person-centred goals.

The current findings also showed that nearly a third of those in Cluster 1 (or nearly a quarter of the total cohort) had NIV initiated when they were clinically stable and had no previous hospitalisations. This reflects the growing interest in outpatient initiation of NIV as a way of improving cost effectiveness.^24^, ^25^, ^26^ A home-based strategy utilising telemedicine has been shown to be non-inferior to an in-hospital approach to NIV initiation in stable hypercapnic COPD with respect to clinical outcomes, and reduced costs by more than 50%.^24^ Our data suggest that the ambulatory approach to NIV initiation is already being implemented in real word practice in France for a significant subgroup of individuals with stable COPD.

Current ATS guidelines recommend screening for obstructive sleep apnoea before the initiation of long-term NIV in stable COPD.^11^ Across Clusters 1–3 in the current analysis, about one-quarter of all individuals underwent sleep apnoea screening with polygraphy or polysomnography in the year before NIV initiation, while only 14% of those in Cluster 4 were screened for sleep apnoea before the start of home NIV therapy. This means that the ATS guidelines were not followed in at least three-quarters of all patients. In addition, it was only really in Cluster 1 that sleep apnoea screening took place a short time before NIV was started (within 1 month). Therefore, there is room for improvement in the use of sleep apnoea screening, and the potential treatment of sleep apnoea with continuous positive airway pressure, prior to initiation of NIV for the management of stable COPD.

One of the key questions with respect to quality of care and health policy relates to the appropriate timing of NIV initiation after an AECOPD, which is currently unclear, even in European and US consensus statements.^10^^,^^11^ It is not yet clear whether hypercapnia should be reassessed several weeks after an acute exacerbation, prior to starting long-term NIV, and there is a significant need for standardisation of follow-up after hospitalisation for AECOPD.^23^ European guidance suggests that an individual should be considered stable when 6 weeks has passed after a COPD-related hospitalisation.^23^ Current ATS guidelines state that long-term NIV should not be initiated during an admission for acute-on-chronic hypercapnic respiratory failure, and suggest reassessment of the NIV requirement at 2–4 weeks after resolution of the acute exacerbation.^11^ However, it has been suggested that clinical history can be used to distinguish between transient acute and acute-on-chronic episodes of hypercapnic respiratory failure during hospitalisation for AECOPD.^27^ Thus, long-term NIV could be initiated during the hospitalisation period for individuals whose history and laboratory findings (e.g. arterial blood gases) are indicative of acute-on-chronic hypercapnic respiratory failure because these individuals will benefit from long-term home NIV.^27^

Another unanswered question relates to how many AECOPD episodes need to occur before a decision is made to start long-term NIV. This is not addressed in current guidelines^10^^,^^11^ and no evidence-based data are available. The different approaches taken in the four clusters identified in the current analysis could be indicative of prescribers’ knowledge and preference. In Cluster 1, prescribers probably had high confidence in the efficacy of long-term NIV and made the therapeutic decision to initiate NIV early and in the ambulatory setting for many individuals. In Clusters 2 and 3, patients underwent more frequent assessments in the year before NIV, prescribers waited for a second or several hospitalisations before being confident that there was an indication for home NIV. Although a very small subset of subjects, those in Cluster 4 had a high number of comorbidities and were hospitalised frequently in the previous year, with an eventual decision to use NIV probably being part of a complex therapeutic plan. Certainly, the significantly higher mortality in Cluster 4, for whom initiation of NIV was quite delayed, suggest that earlier identification of individuals suitable for home NIV and prompt initiation of therapy might be beneficial.

Similar to other recent studies,^28^^,^^29^ a key strength of the current analysis is the dataset used. The French SNDS is a large unbiased claims database that covers 99% of the French population. It includes public and private practices, and in-hospital and ambulatory NIV initiation, and is not specific to any healthcare provider, insurer or device. In addition, the sample size is by far the largest compared with any previous study of NIV in COPD. Another important point is that the entire study period took place before the start of the COVID-19 pandemic, meaning that there is no confusion between NIV initiated for COPD and NIV required to manage COVID-19-related illness.

Several limitations also need to be taken into account when interpreting the study findings. Firstly, the database was designed for administration rather than research purposes and may therefore not contain information on some relevant parameters. For example, due to French privacy requirements, the SNDS does not contain data on smoking habits, alcohol intake or body mass index. In addition, it is important to note that stringent methodology was applied to specifically include only severe AECOPD that required hospitalisation in the current analysis. This is because severe AECOPD requiring hospitalisation can be determined objectively, whereas it is more difficult to identify exacerbations not requiring hospitalisation with consistency and acceptable reliability. In that context, previous data suggest that moderate AECOPD may not be accurately identified by healthcare utilization-based algorithms.^30^ Furthermore, severe exacerbations are particularly important in COPD because these are a major driver of direct costs.^31^

Overall, the findings of this study indicate that the current lack of robust data and strong guideline recommendations results in significant heterogeneity regarding when and for whom long-term, at-home NIV therapy is used to manage COPD. In addition, the heterogeneity of the disease that was evident in the current analysis, and the differing mortality outcomes between the four identified clusters, highlight the importance of personalised strategies for disease management, including the initiation of NIV, based on the previous disease trajectory. It would also be interesting to determine whether other hard clinical outcomes after initiation of NIV (e.g. exacerbations and hospitalisations) vary between these clusters. It would also be helpful to document NIV therapy termination rates and determine the impact of NIV on health-related costs in the different clusters. This knowledge, and the current study findings, could then be used to achieve more consistent, evidence-based and personalised use of home NIV in stable COPD, with the ultimate goal of improving individual patient outcomes.

## Contributors

Study procedures and analyses were undertaken by independent third parties, HEVA (PL, HD, AP) and INSERM HP2 laboratory and artificial intelligence chair “trajectories medicine” (director and principal investigator, JLP). The first draft of the manuscript was prepared by JLP, with the assistance of an independent medical writer funded by ResMed. The manuscript was reviewed and edited by all the authors. All authors made the decision to submit the manuscript for publication.

## Data sharing statement

The data that support the findings of this study are under restricted access, granted by the Comité éthique et scientifique pour les recherches, les études et les évaluations dans le domaine de la santé (CESREES; ref: 3904033) and from the French data protection authority (Comité National de l’Informatique et des Libertés, CNIL), and therefore are not publicly available.

## Declaration of interests

JLP has received lecture fees or conference travel grants from ResMed, Philips, AstraZeneca, Jazz Pharmaceuticals, Agiradom and Bioprojet, and has received unrestricted research funding from ResMed, Philips, GlaxoSmithKline, Bioprojet, Fondation de la Recherche Medicale (Foundation for Medical Research), Direction de la Recherche Clinique du CHU de Grenoble (Research Branch Clinic CHU de Grenoble), and fond de dotation “Agir pour les Maladies Chroniques” (endowment fund “Acting for Chronic Diseases”). FL and AJ are employees of ResMed. PL, HD and AP are employees of HEVA and their participation in this study was funded by ResMed. APa has received consulting fees from ResMed. APr has received investigator fees for clinical trials funded by ResMed. SB has no conflicts of interest to disclose.
