# Supplementary material for: Health trajectories before initiation of non-invasive ventilation for chronic obstructive pulmonary disease: a French nationwide database analysis
Source: Lancet Reg Health Eur. 2023 Aug 29;34:100717. doi: 10.1016/j.lanepe.2023.100717 (PMC10625021; doi:10.1016/j.lanepe.2023.100717)
Supplement: Supplementary Material [file mmc1.docx]

**ONLINE SUPPLEMENT**

**Health trajectories before initiation of non-invasive ventilation for chronic obstructive pulmonary disease: a French nationwide database analysis**

Jean-Louis Pepin^1^, Pauline Lemeille^2^, Hélène Denis^2^, Anne Josseran^3^, Florent Lavergne^3^, Arnaud Panes^2^, Sébastien Bailly^1^, Alain Palot^4^, Arnaud Prigent^5^, on behalf of the medXcloud group

^1^University Grenoble Alpes, Inserm, CHU Grenoble Alpes, HP2, Grenoble, France

^2^HEVA, Lyon, France

^3^ResMed Science Center, Saint-Priest, France

^4^Hôpital Saint-Joseph, Marseille, France

^5^Polyclinique Saint-Laurent, Rennes, France

**Supplementary Methods**

**Methodology for the identification of the COPD cohort and variables of interest**

*Identifying individuals with COPD*

Patients were considered to have chronic obstructive pulmonary disease (COPD) if at least one of the following criteria was met over the 5-year period prior to the index date (which was defined as the first delivery of non-invasive ventilation (NIV) in the study inclusion period – 1 January 2015 to 31 December 2019):

- presence of at least one hospitalisation with relevant ICD-10 codes; OR
- presence of long-term disease related to COPD; AND
- treatment with long-acting β-agonist/long-acting muscarinic antagonist combinations and/or inhaled corticosteroids (before or after initiation of NIV).

| **ICD-10 code** | **Code description label** |
| --- | --- |
| [](https://www.ameli.fr/accueil-de-la-ccam/trouver-un-acte/fiche-abregee.php?code=LDDA001)[](https://www.ameli.fr/accueil-de-la-ccam/trouver-un-acte/fiche-abregee.php?code=LECA001)[](https://www.ameli.fr/accueil-de-la-ccam/trouver-un-acte/fiche-abregee.php?code=LECA006)[](https://www.ameli.fr/accueil-de-la-ccam/trouver-un-acte/fiche-abregee.php?code=LECC001)[](https://www.ameli.fr/accueil-de-la-ccam/trouver-un-acte/fiche-abregee.php?code=LFCA004)J41 | Simple and mucopurulent chronic bronchitis |
| J42 | Unspecified chronic bronchitis |
| J43 | Emphysema |
| J44 | Other chronic obstructive pulmonary disease |
| J961 | Chronic Respiratory Failure |

*COPD exacerbations*

The following combinations were used to identify exacerbations based on INVS publication (Fuhrman C, Delmas MC. Hospitalisations pour exacerbations de BPCO : comment les identifier à partir des données du programme de médicalisation des systèmes d’information ? Saint-Maurice (Fra) : Institut de veille sanitaire, août 2009, 16 p. Available from: http://www.codage.ext.cnamts.fr/codif/tips/index_presentation.php?p_site=AMELI%22).

Hospitalisations with the following ICD-10 codes as the principal diagnosis (PD) and/or secondary associated diagnosis (SAD) were considered as COPD exacerbations:

| PD=J43* |
| --- |
| PD =J44* |
| SAD=J440 |
| SAD=J441 |
| PD or SAD =J960 AND DAS=(J43* or J44*) |
| PD or SAD = (J10-J22) AND (DAS=J43* or J44*) |

| **ICD-10 code** | **Code description label** |
| --- | --- |
| [](https://www.ameli.fr/accueil-de-la-ccam/trouver-un-acte/fiche-abregee.php?code=LDDA001)[](https://www.ameli.fr/accueil-de-la-ccam/trouver-un-acte/fiche-abregee.php?code=LECA001)[](https://www.ameli.fr/accueil-de-la-ccam/trouver-un-acte/fiche-abregee.php?code=LECA006)[](https://www.ameli.fr/accueil-de-la-ccam/trouver-un-acte/fiche-abregee.php?code=LECC001)[](https://www.ameli.fr/accueil-de-la-ccam/trouver-un-acte/fiche-abregee.php?code=LFCA004)J41 | Simple and mucopurulent chronic bronchitis |
| J42 | Unspecified chronic bronchitis |
| J43 | Emphysema |
| J44 | Other chronic obstructive pulmonary disease |
| J440 | Chronic obstructive pulmonary disease with acute lower respiratory infection |
| J441 | Chronic obstructive pulmonary disease with acute exacerbation, unspecified |
| J449 | Chronic obstructive pulmonary disease, unspecified |
| J960 | Acute respiratory failure |
| J961 | Chronic Respiratory Failure |
| J10 | Influenza due to other identified influenza virus |
| J11 | Influenza, virus not identified |
| J12 | Viral pneumonia, not elsewhere classified |
| J13 | Pneumonia due to Streptococcus pneumoniae |
| J14 | Pneumonia due to Haemophilus influenzae |
| J15 | Bacterial pneumonia, not elsewhere classified |
| J16 | Pneumonia due to other infectious organisms, not elsewhere classified |
| J17 | Pneumonia in diseases classified elsewhere |
| J18 | Pneumonia, organism unspecified |
| J20 | Acute bronchitis |
| J21 | Acute bronchiolitis |
| J22 | Unspecified acute lower respiratory infection |

*Hypertension or cardiovascular diseases*

Algorithms from CNAM mapping (Caisse National d'Assurance Maladie. Méthodologie médicale de la cartographie des pathologies et des dépenses, version G8 [années 2015 à 2019, Tous Régimes]. Updated on July 2021. Available from: https://assurance-maladie.ameli.fr/sites/default/files/2021_methode-reperage-pathologies_cartographie_0.pdf) were used to identify relevant medical history of hypertension and/or cardiovascular diseases during 5 years before the index date (pathologies 1, 2, 3, 4, 5, 6, 7, 8, 9, 10, 11, 12).

*Diabetes*

Patients were considered to have diabetes if any of the following disease or medication codes were recorded over the 5-year period prior to the index date. CNAM mapping was also applied.

| **ICD-10 code** | **Code description label** |
| --- | --- |
| \| [](https://www.ameli.fr/accueil-de-la-ccam/trouver-un-acte/fiche-abregee.php?code=LDDA001)[](https://www.ameli.fr/accueil-de-la-ccam/trouver-un-acte/fiche-abregee.php?code=LECA001)[](https://www.ameli.fr/accueil-de-la-ccam/trouver-un-acte/fiche-abregee.php?code=LECA006)[](https://www.ameli.fr/accueil-de-la-ccam/trouver-un-acte/fiche-abregee.php?code=LECC001)[](https://www.ameli.fr/accueil-de-la-ccam/trouver-un-acte/fiche-abregee.php?code=LFCA004)E10 \| \| --- \| | Type 1 diabetes mellitus |
| E11 | Type 2 diabetes mellitus |
| E12 | Malnutrition-related diabetes mellitus |
| E13 | Other specified diabetes mellitus |
| E14 | Unspecified diabetes mellitus |
| G590 | Diabetic mononeuropathy |
| G632 | Diabetic polyneuropathy |
| G730 | Myasthenic syndromes in endocrine diseases |
| G990 | Autonomic neuropathy in endocrine and metabolic |
| H280 | Diabetic cataract |
| H360 | Diabetic retinopathy |
| I792 | Peripheral angiopathy in diseases classified elsewhere |
| L97 | Ulcer of lower limb, not elsewhere classified |
| M142 | Diabetic arthropathy |
| M146 | Neuropathic arthropathy |
| N083 | Glomerular disorders in diabetes mellitus |

| **ATC medication code** |  |
| --- | --- |
| A10 | Insulins and analogues |

*Undernutrition*

Individuals who had at least one hospitalisation with one of the following ICD-10 codes during the 5-year period prior to the index date were considered to be undernourished.

| **ICD-10 code** | **Code description label** |
| --- | --- |
| E40 | Kwashiorkor |
| E41 | Nutritional marasmus |
| E42 | Marasmic kwashiorkor |
| E43 | Unspecified severe protein-energy malnutrition |
| E44 | Protein-energy malnutrition of moderate and mild degree |
| E45 | Retarded development following protein-energy malnutrition |
| E46 | Unspecified protein-energy malnutrition |
| R64 | Cachexie |
| B222 | HIV disease resulting in encephalopathy |

*Morbid obesity*

Individuals who had at least one hospitalisation with one of the following ICD-10 or medical procedure codes (CCAM classification; specific codes edited by the national public insurance system) associated with obesity, OR those treated with continuous positive airway pressure (based on reimbursement under specific follow-up pathways with a home care provider (LPP classification: http://www.codage.ext.cnamts.fr/codif/tips/index_presentation.php?p_site=AMELI")

| **ICD-10 code** | **Code description label** |
| --- | --- |
| E66 | Obesity |
| E662 | Extreme obesity with alveolar hypoventilation |

| **CCAM code** | **Code description label [in French]** |
| --- | --- |
| HFCA001 | Court-circuit gastrique avec anse montée en Y [Bypass gastrique en Y] pour obésité morbide, par laparotomie |
| HFCC003 | Court-circuit gastrique avec anse montée en Y [Bypass gastrique en Y] pour obésité morbide, par coelioscopie |
| HFKC001 | Changement d'un anneau ajustable périgastrique pour obésité morbide, par coelioscopie |
| HFKA002 | Changement d'un anneau ajustable périgastrique pour obésité morbide, par laparotomie |
| HFFC018 | Gastrectomie longitudinale [Sleeve gastrectomy] pour obésité morbide, par coelioscopie |
| HFFA011 | Gastrectomie longitudinale [Sleeve gastrectomy] pour obésité morbide, par laparotomie |
| HFMA010 | Gastroplastie verticale calibrée pour obésité morbide, par laparotomie |
| HFMC006 | Gastroplastie verticale calibrée pour obésité morbide, par coelioscopie |
| HGCA009 | Court-circuit biliopancréatique ou intestinal pour obésité morbide, par laparotomie |
| HGCC027 | Court-circuit biliopancréatique ou intestinal pour obésité morbide, par coelioscopie |
| HFMC007 | Gastroplastie par pose d'anneau ajustable périgastrique pour obésité morbide, par coelioscopie |
| HFMA009 | Gastroplastie par pose d'anneau ajustable périgastrique pour obésité morbide, par laparotomie |
| HFGC900 | Ablation d'une sonde de stimulation pariétale gastrique pour obésité morbide, par coelioscopie |
| HFLE002 | Pose d'un ballonnet intragastrique pour obésité morbide, par oeso-gastro-duodénoscopie |
| HFFA001 | Gastrectomie avec court-circuit biliopancréatique ou intestinal pour obésité morbide, par laparotomie |
| HFFC004 | Gastrectomie avec court-circuit biliopancréatique ou intestinal pour obésité morbide, par coelioscopie |
| HFKA001 | Changement ou repositionnement du dispositif d'accès d'un anneau ajustable périgastrique pour obésité morbide, par abord direct |
| HFLC900 | Implantation sous-cutanée d'un stimulateur gastrique avec pose d'une sonde pariétale gastrique pour obésité morbide, par coelioscopie |

*Sleep apnea*

Individuals were considered to have sleep apnea if the following ICD-10 code indicating sleep apnea was recorded during the 5-year period prior to the index date.

| **ICD-10 code** | **Code description label** |
| --- | --- |
| G473 | Sleep apnoea |

*Psychiatric diseases, anxiety or depression*

Individuals were considered to have any of these conditions if there was at least one hospitalisation with one of the following ICD-10 codes and/or if there was at least two deliveries/reimbursements for one of the listed medications during the 5-year period prior to the index date. For depression, CNAM mapping algorithms were used.

| **ICD-10 code** | **Code description label** |
| --- | --- |
| F20 | Schizophrenia |
| F21 | Schizotypal disorder |
| F22 | Persistent delusional disorders |
| F23 | Acute and transient psychotic disorders |
| F24 | Induced delusional disorder |
| F25 | Schizoaffective disorders |
| F28 | Other nonorganic psychotic disorders |
| F29 | Unspecified nonorganic psychosis |
| F30 | Manic episode |
| F31 | Bipolar affective disorder |
| [](https://www.ameli.fr/accueil-de-la-ccam/trouver-un-acte/fiche-abregee.php?code=LDDA001)[](https://www.ameli.fr/accueil-de-la-ccam/trouver-un-acte/fiche-abregee.php?code=LECA001)[](https://www.ameli.fr/accueil-de-la-ccam/trouver-un-acte/fiche-abregee.php?code=LECA006)[](https://www.ameli.fr/accueil-de-la-ccam/trouver-un-acte/fiche-abregee.php?code=LECC001)[](https://www.ameli.fr/accueil-de-la-ccam/trouver-un-acte/fiche-abregee.php?code=LFCA004)F32 | Depressive episode |
| F33 | Recurrent depressive disorder |
| F34 | Persistent mood [affective] disorders |
| F38 | Other mood [affective] disorders |
| F39 | Unspecified mood [affective] disorder |
| F40 | Phobic anxiety disorders |
| F41 | Other anxiety disorders |
| F42 | Obsessive-compulsive disorder |
| F43 | Reaction to severe stress, and adjustment disorders |
| F44 | Dissociative [conversion] disorders |
| F45 | Somatoform disorders |

| **ATC medication code** |  |
| --- | --- |
| N05B | Tranquilizers/anti-anxiety drugs |
| N06A | Anti-depressant |
| N03 | Anti-epileptics |
| N04 | Anti-Parkinson drugs |
| N05 | Psycholeptics |
| N06 | Psychoanaleptics |

**TAK analysis**

The clustering workflow in the TAK methodology is comparable to classical methods of sequence analysis such as TraminR (that can also run HCA based on Hamming distance and Ward linkage method; Gabadinho A, et al. J Stat Software 2011;40(4):1-37). The strength of the TAK methodology is mainly that it provides greater flexibility for the visualization of clusters with editing capabilities, including a zooming function, to increase readability. Furthermore, the TAK methodology enables better management of the visualization of gaps (time delay) between events. In our dataset, exacerbations/ hospitalizations were relatively short duration events to manage, and this is why the TAK methodology was deemed to be appropriate. Another strength of the TAK method is that it deals with a dependency between events. The events considered here were: all hospitalisations, hospitalisations in cardiology, hospitalisations in intensive care, and COPD exacerbations. Finite mixture models such as proc traj in SAS initially only allow consideration of trajectory evolution for one outcome. An extension was proposed to consider the trajectories of several outcomes, but this requires these outcomes to have independent distributions. This requirement for independence of distribution of outcomes is not plausible for our dataset. However, we have performed a clustering using finite mixture modelling (proc traj), which showed similar trajectories to the current analysis (work presented at the EMOIS congress in French: <https://static>.hevaweb.com/web/PDF/fed4bfc774146-com-resmed-montana-emois-2023.pdf). To our knowledge there is no easy way to formally compare the performance of classification of finite mixture models (selected using AIC criteria) and hierarchical cluster analysis (HCA).

For TAK modelisation, we chose the Hamming distance for two reasons: first, for its robustness to small discrepancies between sequences from two different patients (e.g., two 365-day-long vectors with one 1-day discrepancy are considered as 99.7% identical) and second, it provides a score based on the vector’s length. HCA methodology is very often used to cluster vectors and to order vector by similarity.

Following the optimal leaf ordering for the hierarchical clustering method developed by Bar-Joseph et al (Bioinformatics 2001;17Suppl:S22-29), we sorted patients in the clustering tree by flipping each node to minimize the previously defined distance between the innermost leaves of two adjacent nodes. This computation simplifies the temporal patterns found in the treatment sequences. Thereby, we obtained a treatment matrix with the time dimension on the x-axis and all patients stacked on the y-axis.

In addition, the final visualization allowed us to keep maximum information despite the very high dimensions. We applied a noise filtering technique to this matrix to only retain meaningful treatment patterns. This technique is a modal filter parametrised by a kernel size, which replaces each value by the most frequent value in the kernel. The kernel size was chosen based on the population size and the expected image output dimensions.

**Figure S1: Flowchart**

**Table S1:** Baseline demographics and characteristics of individuals with initiation of non-invasive ventilation without any prior hospitalisation or exacerbation compared with the total cohort

|  | **No previous hospitalisation or exacerbation (n=11,405)** | **Total cohort  (n=54,545)** | **p-value** |
| --- | --- | --- | --- |
| Age, years | 68 (60, 76) | 70 (62, 79) | <0·001 |
| Male sex, n (%) | 5,932 (52) | 27,920 (51) | Ns |
| Number of exacerbations requiring hospitalisation in the year before NIV initiation | 0 (0, 0) | 1 (0, 2) | <0·001 |
| No exacerbations requiring hospitalisation in the year before NIV initiation | 11,405 (100) | 23,024 (42) | <0·001 |
| Medical history within the previous 5 years, n (%) |  |  |  |
| Undernutrition | 1,305 (11) | 15,140 (28) | <0·001 |
| Morbid obesity | 4,991 (44) | 25,158 (46) | <0·001 |
| Sleep apnoea | 4,338 (38) | 18,309 (34) | <0·001 |
| Hypertension or cardiovascular disease | 6,976 (61) | 41,262 (76) | <0·001 |
| Diabetes | 3,453 (30) | 16,137 (30) | <0·001 |
| Psychiatric diseases, anxiety or depression | 3,663 (32) | 20,338 (37) | <0·001 |
| Charlson comorbidity index n (%) |  |  |  |
| 0 | 1,054 (9) | 3,889 (7) | <0·001 |
| 1–2 | 9,425 (83) | 42,572 (78) | <0·001 |
| 3–4 | 896 (8) | 7,591 (14) | <0·001 |
| ≥5 | 30 (<1) | 493 (1) | <0·001 |

Values are median (interquartile range) or number of patients (%).

AECOPD, acute exacerbations of chronic obstructive pulmonary disease; NIV, non-invasive ventilation; ns, not statistically significant.

**Table S2:** Investigations and consultations in the year before the initiation of non-invasive ventilation for individuals with initiation of non-invasive ventilation without any prior hospitalisation or exacerbation compared with the total cohort

|  | **No previous hospitalisation or exacerbation (n=11,405)** | **Total cohort (n=54,545)** |  |
| --- | --- | --- | --- |
| Sleep studies (PG or PSG) |  |  |  |
| 0 | 6,680 (59) | 40,765 (75) | <0·001 |
| 1–2 | 4,275 (37) | 12,516 (23) |  |
| 3–5 | 450 (4) | 1,116 (2) |  |
| Pulmonary function tests |  |  |  |
| 0 | 3,162 (28) | 20,335 (37) | <0·001 |
| 1–2 | 6.571 (58) | 25,624 (47) |  |
| 3–5 | 1,553 (14) | 7,313 (13) |  |
| ≥6 | 119 (1) | 1,273 (2) |  |
| Hospital consultations |  |  |  |
| 0 | 5,121 (45) | 24,442 (45) | <0·05 |
| 1–2 | 2,986 (26) | 14,943 (27) |  |
| 3–5 | 1,939 (17) | 8,836 (16) |  |
| ≥6 | 1,359 (12) | 6,324 (12) |  |
| Pulmonologist consultations |  |  |  |
| 0 | 9.084 (80) | 47,572 (87) | <0·001 |
| 1–2 | 2,030 (18) | 5,930 (11) |  |
| ≥3 | 291 (3) | 1,043 (2) |  |
| General practitioner consultations |  |  |  |
| 0 | 410 (4) | 2,491 (5) | <0·001 |
| 1–2 | 530 (5) | 3,106 (6) |  |
| 3–5 | 2,263 (20) | 9,960 (18) |  |
| 6–10 | 3,981 (35) | 17,602 (32) |  |
| 11-20 | 3,419 (30) | 16,816 (31) |  |
| >20 | 802 (7) | 4,570 (8) |  |

Values are number of patients (%).

PG, polygraphy; PSG, polysomnography.

**Table S3:** Pairwise comparison of mortality risk in different patient clusters

| **Group** | **Reference** | **Hazard ratio** | **95% confidence interval** | **p-value** |
| --- | --- | --- | --- | --- |
| Cluster 2 | Cluster 1 | 1.468 | 1.410; 1.528 | <0.0001 |
| Cluster 3 | Cluster 1 | 1.602 | 1.551; 1.655 | <0.0001 |
| Cluster 4 | Cluster 1 | 2.693 | 2.445; 2.966 | <0.0001 |
| Cluster 3 | Cluster 2 | 1.091 | 1.044; 1.141 | 0.0001 |
| Cluster 4 | Cluster 2 | 1.834 | 1.658; 2.029 | <0.0001 |
| Cluster 4 | Cluster 3 | 1.681 | 1.524; 1.854 | <0.0001 |

**Table S4:** Mortality rates in patient subgroups (multivariable Cox regression analysis)

| **Variable** | **Class** | **Reference category** | **Total N** | **Proportion of events** | **HR (95% CI)** | **p-value** | **Global p-value** |
| --- | --- | --- | --- | --- | --- | --- | --- |
| Age group  (years) | 45-54 | 35-44 | 4,465 | 81.32% | 1.09 (0.90–1.31) | 0.3908 | <0.0001 |
|  | 55-64 |  | 12,107 | 74.24% | 1.51 (1.26– 1.80) | <0.0001 |  |
|  | 65-74 |  | 16,905 | 65.38% | 2.15 (1.80–2.57) | <0.0001 |  |
|  | 75+ |  | 20,326 | 47.07% | 3.87 (3.24–4.61) | <0.0001 |  |
| Sex | Male | Female | 27,920 | 59.95% | 1.21 (1.17–1.24) | <0.0001 | <0.0001 |
| Trajectory before NIV initiation | Cluster 2 | Cluster 1 | 6,543 | 53.52% | 1.47 (1.41–1.53) | <0.0001 | <0.0001 |
|  | Cluster 3 |  | 11,375 | 51.08% | 1.60 (1.55–1.66) | <0.0001 |  |
|  | Cluster 4 |  | 652 | 33.90% | 2.69 (2.44–2.97) | <0.0001 |  |
| Charlson score | 1-2 | 0 | 42,572 | 63.82% | 1.07 (1.01–1.13) | 0.0274 | <0.0001 |
|  | 3-4 |  | 7,591 | 50.65% | 1.33 (1.24–1.42) | <0.0001 |  |
|  | 5-6 |  | 488 | 37.09% | 1.68 (1.48–1.91) | <0.0001 |  |
|  | 7+ |  | 5 | 40.00% | 1.00 (0.32–3.10) | 0.9988 |  |

CI, confidence interval; HR, hazard ratio; N, number; NIV, non-invasive ventilation.

**MedXcloud group:**

- Pr. Jean-Louis Pépin, University Grenoble Alpes, Inserm, CHU Grenoble Alpes, HP2, Grenoble, France
- Pr. Atul Malhotra, University of California San Diego, San Diego, CA, USA
- Pr. Peter Cistulli, Charles Perkins Centre, Faculty of Medicine and Health, University Sydney; and Department of Respiratory Medicine, Royal North Shore Hospital, Sydney, NSW, Australia
- Adam Benjafield, ResMed Science Center, Sydney, NSW, Australia
- Kimberly Sterling, ResMed Science Center, San Diego, CA, USA
